# Thoracoscopic resection of torsion of the right middle lobe after right lower lobectomy: A case report

**DOI:** 10.1016/j.xjtc.2025.10.006

**Published:** 2025-10-24

**Authors:** Sota Yoshimine, Yosuke Matsuura, Mitsue Kawahara, Ayumi Suzuki, Junji Ichinose, Masayuki Nakao, Mingyon Mun

**Affiliations:** Department of Thoracic Surgical Oncology, Cancer Institute Hospital, Japanese Foundation for Cancer Research, Tokyo, Japan

**Figure:**
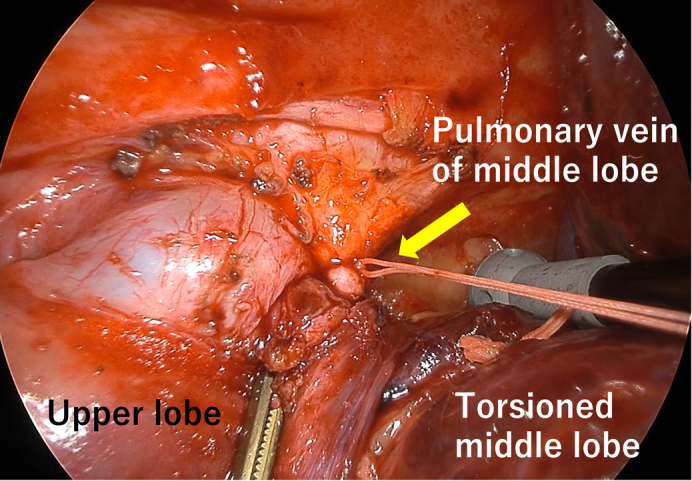
First, divide the pulmonary vein when resecting a torted pulmonary lobe.

**Central Message**

Middle lobe torsion after right lower lobectomy is rare but must be considered in patients with well-lobulated fissures and extensive hilar dissection.

Lobar torsion is a potentially fatal complication of pulmonary resection, with an incidence of 0.089% to 0.3%.^1^ Middle lobe torsion (MLT) after right lower lobectomy (RLL) is extremely rare, with no cases in a review of 109 reports.^2^ When resecting a torted lobe, dividing the pulmonary vein (PV) before detorsion, or resecting without detorsion, is recommended to avoid embolization of necrotic material or thrombus.^2^ We report a case of MLT after thoracoscopic RLL treated with thoracoscopic middle lobectomy, emphasizing the PV division.

## Case Presentation

The study was approved by the institutional review board (No.: 2023-GB-078; October 11, 2023) and the patient provided written consent for publication.

**Figure 1:**
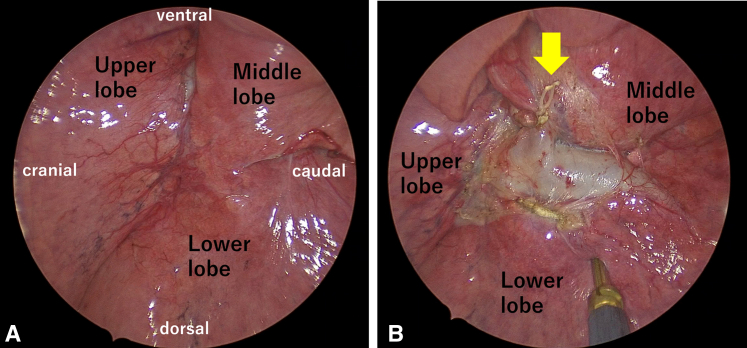
Initial intraoperative view. A, Well-lobulated major and minor fissures. B, Parenchymal bridge is ligated and divided (*arrow*).

A 53-year-old woman with cT1 bN0 M0, IA2 lung cancer underwent thoracoscopic RLL with systematic lymph node dissection. Well-lobulated fissures were present (Figure 1, *A*), and the minimal parenchymal bridge between the middle lobe (ML) and lower lobe was ligated with silk on the ML side and divided (Figure 1, *B*). The caudal ML bronchus and PV were mobilized distally for easy and accurate mediastinal lymph node dissection. The ML initially expanded normally (Video 1). The patient was discharged on postoperative day (POD) 5.

**Figure 2:**
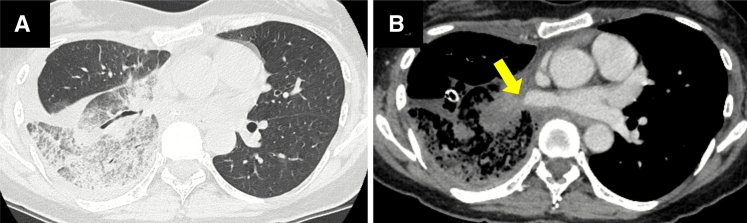
Computed tomography (*CT*) images. A, Localized consolidation in the middle lobe and pleural effusion. B, Contrast CT image showing interruption of the middle lobe pulmonary vein (*arrow*).

On POD 8, she developed cough and hemoptysis and presented on POD 10 with fever, leukocytosis (14 430/mm^3^), and elevated C-reactive protein (12.68 mg/dL). Computed tomography (CT) revealed consolidation of the ML and pleural effusion (Figure 2, *A*). Antibiotics and drainage were initiated. On POD 13, a high-grade fever developed, and contrast-enhanced CT demonstrated interruption of the ML PV (Figure 2, *B*). Emergency surgery was performed, and MLT was diagnosed.

**Figure 3:**
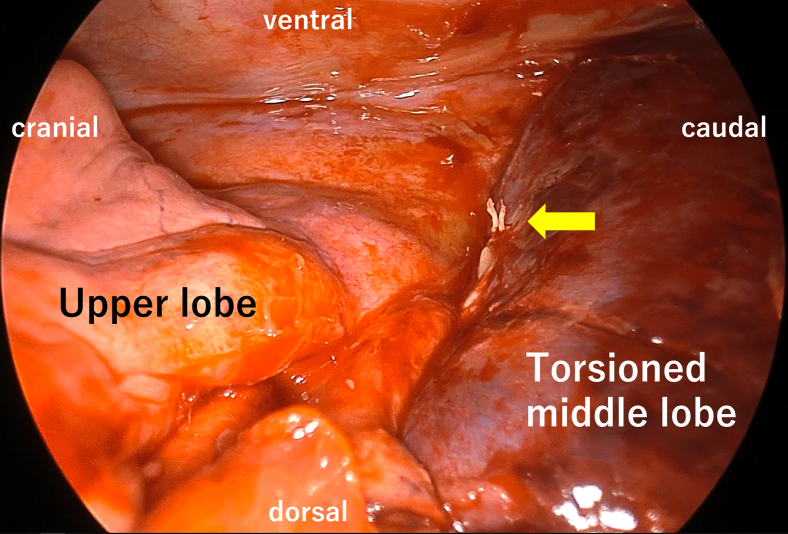
Intraoperative view at reoperation: Enlarged, dark middle lobe with silk ligature located ventrally (*arrow*), consistent with 90° clockwise torsion.

Intraoperatively, bronchoscopy revealed an obstruction of the ML bronchus. The ML was enlarged and dark red, resembling liver tissue. The silk ligature was located ventrally, indicating a 90° clockwise torsion (Figure 3). The inflamed mediastinal tissue around the upper PV was dissected to expose the bifurcation, and the ML PV was encircled; however, an adequate length for stapling was not obtainable. The proximal PV was double ligated and divided. Sequential resection of the twisted parenchyma exposed the pulmonary artery (PA). The lateral ML PA branch was exposed during the previous surgery and bled. Therefore, only the medial ML PA branch was initially cut, and the distal PV was ligated. The lateral ML PA branch and bronchus were stapled, and the lobe was removed thoracoscopically (Video 2). The operative time was 3 hours 44 minutes, with blood loss of 365 mL (including pleural effusion). The patient recovered uneventfully and was discharged 8 days after the reoperation.

## Discussion

MLT after RLL is extremely rare, and in reported series diagnosis typically occurs around POD 3; our case presented late.^2^ Factors likely contributing to the torsion included well-lobulated fissures between the upper and middle lobes, leaving little adhesion; distal mobilization of the ML PV during lymphadenectomy, reducing vascular anchoring; and wide hilar dissection, diminishing support. Despite initial normal expansion, the loss of anatomical support likely permitted ventral rotation.

Diagnostic delay increases the risks of infarction, sepsis, and acute respiratory distress syndrome.^2^ In cases of early or partial torsion, detorsion may be attempted. However, a swollen, dark lobe is usually nonviable and requires resection. PV division before detorsion or resection minimizes embolic risk; however, our case demonstrated that inflammation and distortion can impede safe PV control. CT showing PV interruption or bronchoscopy narrowing is useful for early diagnosis.^2^^,^^3^

A thoracoscope with a zoomed-in view was used to perform ML resection. Open thoracotomy would have shortened the operative time, but it would have been more invasive. Because this ML resection followed RLL, avoiding damage to the respiratory muscles was important. Although thoracoscopic surgery succeeded here, surgeons must be prepared to convert to thoracotomy if bleeding or poor visualization compromise safety.

## Conclusions

Pulmonary torsion can rapidly progress to infarction and systemic complications. Early diagnosis and surgical management are essential. Although MLT after RLL is rare, it is possible in patients with well-lobulated fissures and extensive hilar dissection.

## Conflict of Interest Statement

The authors reported no conflicts of interest.

The *Journal* policy requires editors and reviewers to disclose conflicts of interest and to decline handling or reviewing manuscripts for which they may have a conflict of interest. The editors and reviewers of this article have no conflicts of interest.

## Supplementary Data

Video 1 Thoracoscopic right lower lobectomy. Video available at: https://www.jtcvs.org/article/S2666-2507(25)00465-1/fulltext.

Video 2 Bronchoscopy and right middle lobectomy via thoracoscopy for the middle lobe torsion. Video available at: https://www.jtcvs.org/article/S2666-2507(25)00465-1/fulltext.
