# Supplementary figures and images for: Thoracoscopic resection of torsion of the right middle lobe after right lower lobectomy: A case report
Source: JTCVS Tech. 2025 Oct 24;35:102130. doi: 10.1016/j.xjtc.2025.10.006 (PMC12881787; doi:10.1016/j.xjtc.2025.10.006)

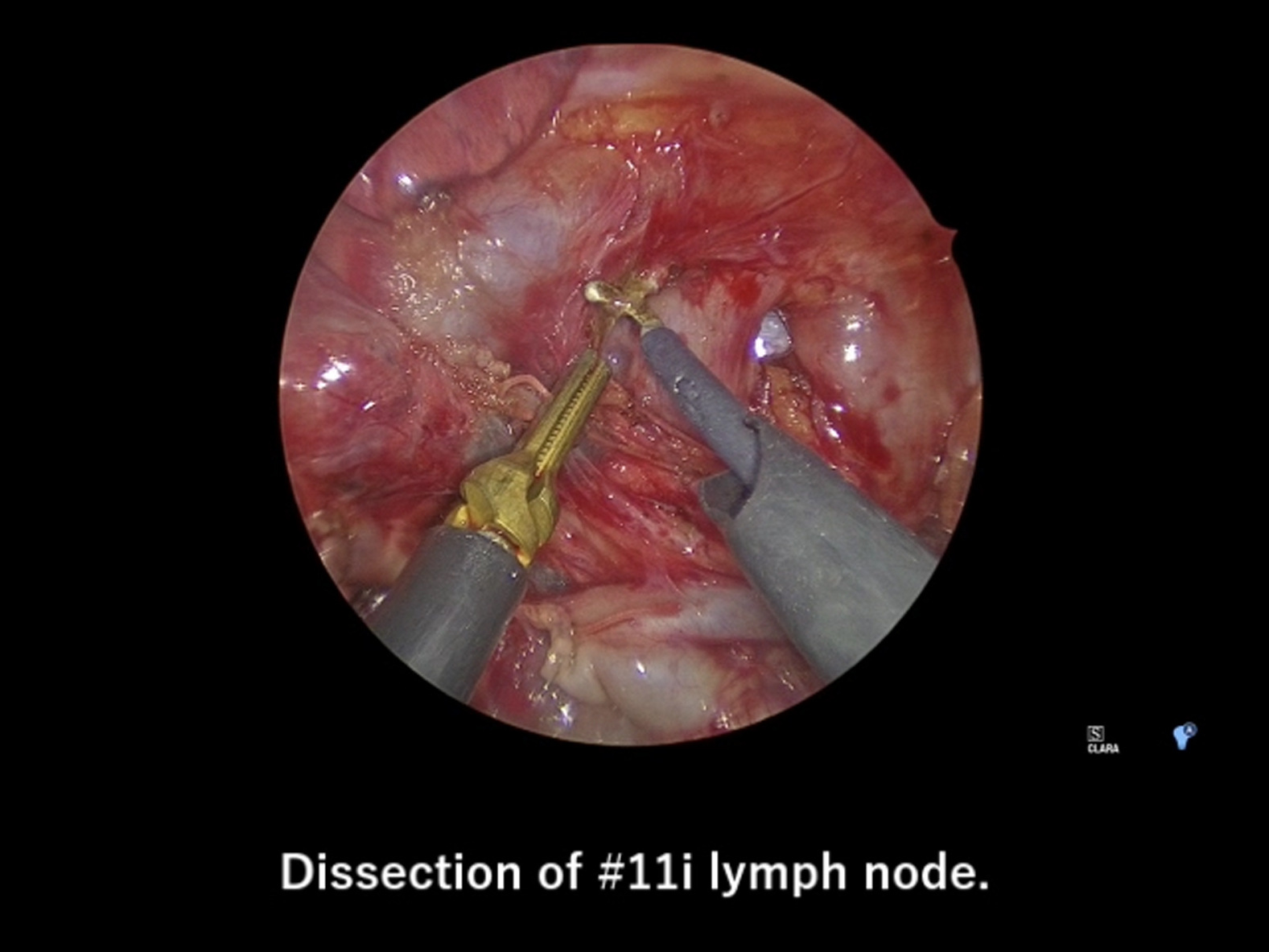

Supplement: Video 1 — Thoracoscopic right lower lobectomy. Video available at: https://www.jtcvs.org/article/S2666-2507(25)00465-1/fulltext. [file fx2.jpg]

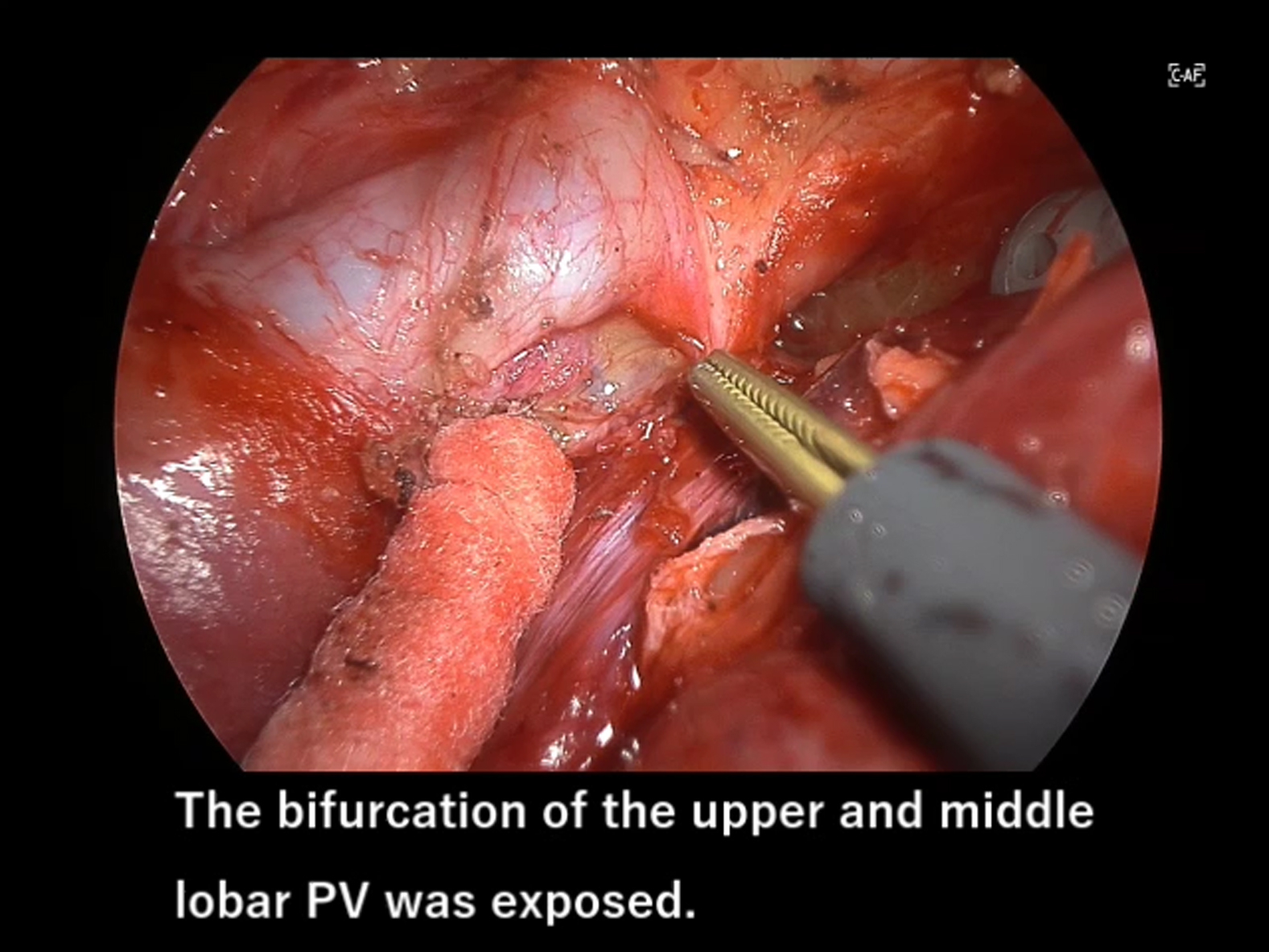

Supplement: Video 2 — Bronchoscopy and right middle lobectomy via thoracoscopy for the middle lobe torsion. Video available at: https://www.jtcvs.org/article/S2666-2507(25)00465-1/fulltext. [file fx3.jpg]
